# Identification of quality gaps in healthcare services using the SERVQUAL instrument and importance-performance analysis in medical intensive care: a prospective study at a medical center in Taiwan

**DOI:** 10.1186/s12913-020-05764-8

**Published:** 2020-09-29

**Authors:** Shu-Ju Lu, Hsiu-O Kao, Bao-Lin Chang, Shu-Ing Gong, Shu-Mei Liu, Shih-Chi Ku, Jih-Shuin Jerng

**Affiliations:** 1 Department of Nursing, National Taiwan University Hospital, No. 7 Zhongshan South Road, Taipei, 10002 Taiwan; 2 Department of Nursing, National Taipei University of Nursing and Health Science, No. 365, Ming-Te Road, Pei-Tou District, Taipei, 11219 Taiwan; 3 Department of Internal Medicine, National Taiwan University Hospital, No. 7 Zhongshan South Road, Taipei, 10002 Taiwan; 4 Center for Quality Improvement, National Taiwan University Hospital, No. 7 Zhongshan South Road, Taipei, 10002 Taiwan

**Keywords:** Intensive care units, Health services, Quality of health care, Expectations, Perception

## Abstract

**Background:**

Assessing patients’ expectations and perceptions of health service delivery is challenging. To understand the service quality in intensive care units (ICUs), we investigated the expected and perceived service quality of ICU care.

**Methods:**

We conducted this study at an ICU of a university-affiliated medical center in Taiwan from April to September 2019. Admitted patients or their family members responded to a questionnaire survey adopted from the SERVQUAL instrument consisting of 22 items in five dimensions. The questionnaire was provided on ICU admission for expectation and before ICU discharge for perception. We analyzed the quality gaps between the surveys and applied important-performance analysis (IPA).

**Results:**

A total of 117 patients were included (62.4% males, average age: 65.9 years, average length of stay: 10.1 days, and 76.9% survival to ICU discharge). The overall weighted mean scores for the surveys were similar (4.57 ± 0.81 and 4.58 ± 0.52, respectively). The ‘tangibles’ dimension had a higher perception than expectation (3.99 ± 0.55 and 4.31 ± 0.63 for expectation and perception, respectively, *p* < 0.001). IPA showed that most of the items in ‘reliability,’ ‘responsiveness’ and ‘assurance’ were located in the quadrant of high expectation and high perception, whereas most of the items in ‘tangibles’ and ‘empathy’ were located in the quadrant of low expectation and low perception. One item (item 1 for ‘tangibles’) was found in the quadrant of high expectation and low perception.

**Conclusions:**

The SERVQUAL approach and IPA might provide useful information regarding the feedback by patients and their families for ICU service quality. In most aspects, the performance of the ICU satisfactorily matched the needs perceived by the patients and their families.

## Background

Measurement of the quality of healthcare is essential for healthcare systems [1, 2]. While a variety of common structure, process, and outcome measurements on the quality of healthcare delivery have been applied [1, 3], one emerging focus is the assessment of the service quality of healthcare by determining how well it meets expectations [4–6]. The introduction of the concept of service quality [7, 8] into the healthcare sector has been an interesting topic. However, assessing the patients’ expectations and perceptions of health service delivery remains challenging. In the past decades, satisfaction surveys have been commonly conducted by healthcare systems to understand the quality from the viewpoint of the patients and visitors, allowing the provider to understand the association between medical service quality and patient satisfaction [9]. Researchers have suggested that the provider should compare the expected and perceived service quality to identify gaps between them [7], apply the feedback from patients to improve the quality of care [10], assess the real experiences of medical care [11], and perceptions of quality as provided by the patients [12]. As service quality has become an important corporate strategy for healthcare organizations [6], measuring perception may be an important approach [13], and understanding the gap between expectation and perception may provide additional insight into the background of traditional satisfaction surveys.

Several reports have discussed this gap measurement approach in assessing healthcare [5, 14, 15], and studies on the service quality of healthcare in Taiwan have also been reported [16–19]; however, reports on service quality in the critical care setting remain limited [20]. The clinical and care settings of intensive care units (ICUs) are characterized by the increased severity of the patients’ illness and the marked abundance of care and treatment modalities, making it difficult for the patients and their family members to provide their opinions about the experience during the patients’ stay. Beyond satisfaction, additional tools for approaching the gap assessment might be needed.

Two currently available tools used to understand expectation and perception in service research are the SERVQUAL instrument [21] and importance-performance analysis (IPA) [22]. The SERVQUAL model is a multi-dimension survey instrument consisting of multiple items proposed by Parasuraman et al. to understand the customers’ expectations and perceptions for service and identify service gaps to improve service [21]. IPA was originally proposed by Martilla and James [22] as a technique for measuring attribute importance and performance to identify service strengths and weaknesses and develop effective marketing programs. With a two-dimensional matrix, IPA uses importance as an X-axis and performance as a Y-axis to categorize items or attributes into four quadrants, and items located in different quadrants may represent different implications for managerial actions [22]. IPA has been used in general practice [23] and pediatric healthcare [12], and the SERVQUAL instrument has been used to measure patients’ opinions of nursing care [16, 24] as well as a variety of hospital services [25–27] and various healthcare environments [19, 28–30]. A newer hierarchical model of health service quality has also been developed [6], contributing to increased academic interest in satisfaction and service quality. There have been reports focusing on specific care settings about the service quality of healthcare; however, few reports from Western or East Asian countries have applied the combination of these two instruments to evaluate service quality for patients in a critical care setting [31, 32]. In our previous work [20], we adopted SERVQUAL and IPA for the settings of the Respiratory Care Center, a dedicated unit for caring long-term ventilator-dependent patients [20]. However, the care setting of ICU, with patients in more acute and critical conditions, might render different perspectives for the patients and their families. Therefore, this study aimed to understand the gaps in service quality in the medical critical care setting to explore opportunities for improvement further, with the research question of ‘What are the significant gaps in service quality of healthcare in the ICU?’

## Methods

### Study design and setting

This was a prospective cross-sectional study conducted from April to September 2019 at a medical ICU of National Taiwan University Hospital, a university-affiliated public medical center in Taiwan. This hospital is a public university-affiliated tertiary medical center consisting of about 2300 beds in northern Taiwan, with about 6400 full-time healthcare workers. The hospital is regulated by the National Health Insurance system, covering more than 95% of expenses. Accordingly, the patients come from all socioeconomic groups and are required to pay only a small portion of the healthcare fee, which has a fixed ceiling amount in the inpatient setting, even for admission to the ICU. The ICU in this study consists of 15 beds, with about 40 patients admitted every month, and cares for patients with a variety of critical medical illnesses. Visitors to the ICU, who are usually family members of the patients, are allowed to visit during three time periods every day in the morning, afternoon, and evening, with each period lasting for 1 h. During the visits, the family members can interact with the on-site healthcare workers and the patient and discuss their condition and planning related to care. The family members can also provide feedback or requests during the visits.

### Participants

The investigators screened all patients upon admission to the ICU. Those who were able to provide consent to participate in the study within 48 h after admission were considered eligible for the study. Patients were excluded if they were younger than 20 years of age, or if they had no surrogate in case they could not participate in the questionnaire.

### The questionnaire

The modified SERVQUAL scale has been shown to have adequate reliability and validity [21]. This instrument was generic, and adoption into the real-world setting was required [21]. In our previous study in another care setting, we had adopted this instrument and demonstrated its internal validity and reliability as translated into Chinese for a domestic setting [20]. In this study, forward translation of the scale into Chinese was performed by two of the study authors (SJL and JSJ) who are knowledgeable about healthcare terminology and the contents of the questionnaire in both English and Chinese. All experts involved in this study discussed the translated Chinese questionnaire to resolve any ambiguities and discrepancies until a general agreement was reached regarding the content. Backward translation of the scale into English was performed by another two physicians of the ICUs of this hospital who were blinded to the questionnaire. The study researchers then compared the original and translated versions, and the Chinese version was further revised and modified until the final version was agreed upon by consensus. The universal agreement score (scale-level content validity index divided by the number of items) was 1.0 [33]. The expectation and perception sections consisted of a structured questionnaire consisting of 22 statements scored using a Likert-type five-point scale in five dimensions (tangibles, reliability, responsiveness, assurance, and empathy). In addition, point-allocation questions were provided [21] (Table 1). In this study, the questionnaire was provided on admission to the ICU for the expectation section, and within 2 days before discharge from the ICU for the perception section and point-allocation for the service quality. In addition to the SERVQUAL items, the questionnaire also obtained the responder’s gender, age, profession, educational level, religion, relationship with the patient in the family, and their prior experience with family members treated at the ICU. The internal consistency of this instrument for each dimension was measured, with Cronbach’s values of 0.932, 0.931, 0.930, 0.930, and 0.930 for the tangibles, reliability, responsiveness, assurance, and empathy dimensions, respectively. In factor analysis, the Kaiser-Meyer-Olkin test value was 0.869, and Bartlett’s Test of Sphericity showed a chi-square of 1436.2 (*p* < 0.001), suggesting good sampling adequacy. Confirmatory factor analysis using the Varimax method [34] showed that the extracted factors contributed to 67.2% of the variance.

**Table 1:** Items of the SERVQUAL scale questionnaire used in this study^a^

Expectation section	Perception section
*Tangibles*	*Tangibles*
E1. The ICU will have modern-looking equipment.	P1. The ICU has modern-looking equipment.
E2. The ICU ‘s physical facilities will be visually appealing.	P2. The ICU’s physical facilities are visually appealing.
E3. The ICU ‘s workers will be neat-appearing	P3. The ICU’s workers are neat-appearing
E4. Materials associated with the service will be visually appealing at the ICU.	P4. Materials associated with the service are visually appealing at the ICU.
*Reliability*	*Reliability*
E5. When the ICU promises to do something by a certain time, it will do so.	P5. When the ICU promises to do something by a certain time, it does so.
E6. When the patient or you have a problem, the ICU will show a sincere interest in solving it.	P6. When the patient or you have a problem, the ICU shows a sincere interest in solving it.
E7. The ICU will perform the service right the first time.	P7. The ICU performs the service right the first time.
E8. The ICU will provide its service at the time it promises to do so.	P8. The ICU provides its service at the time it promises to do so.
E9. The ICU will insist on error-free records.	P9. The ICU insists on error-free records.
*Responsiveness*	*Responsiveness*
E10. Workers at the ICU will tell you exactly when the care will be performed.	P10. Workers at the ICU tell you exactly when the care will be performed.
E11. Workers at the ICU will give the patient prompt care.	P11. Workers at the ICU give the patient prompt care.
E12. Workers at the ICU will always be willing to help you and the patient.	P12. Workers at the ICU are always willing to help you and the patient.
E13. Workers at the ICU will never be too busy to respond to the patient’s or your requests.	p13. Workers at the ICU are never too busy to respond to the patient’s or your requests.
*Assurance*	*Assurance*
E14. The behavior of the workers at the ICU will instill confidence in the patient and the family.	P14. The behavior of the workers at the ICU instills confidence in the patient and the family.
E15. You will feel safe for the patient’s care by the ICU.	P15. You feel safe for the patient’s care by the ICU.
E16. Workers at the ICU will be consistently courteous with the patient and the family.	P16. Workers at the ICU are consistently courteous with the patient and the family.
E17. Workers at the ICU will have the knowledge to answer your questions.	P17. Workers at the ICU have the knowledge to answer your questions.
*Empathy*	*Empathy*
E18. The ICU will give you and the patient individual attention.	P18. The ICU gives you and the patient individual attention.
E19. The ICU will have operating hours convenient to its patients and families.	P19. The ICU has operating hours convenient to its patients and families.
E20. The ICU will have workers who give the patient and the family personal attention.	P20. The ICU has workers who give the patient and the family personal attention.
E21. The ICU will have the best interest of the patient and the family at heart.	P21. The ICU has the best interest of the patient and the family at heart.
E22. Workers at the ICU will understand the special needs of the patient and the family.	P22. Workers at the ICU understand the special needs of the patient and the family.
**Point-allocation questions**	
1. The appearance of the ICU’s facilities, equipment, the workers and the materials used.	
2. The ability of this ICU to perform promised services dependably and accurately.	
3. The willingness of the ICU to help patients and families and provide prompt service.	
4. The knowledge and courtesy of the ICU’s workers and their ability to convey trust and confidence.	
5. The caring, individualized attention the ICU provides the patients and families.	

*ICU* Intensive care unit

^a^Actual questionnaire was provided in Chinese; this is a translation of the questionnaire form

An investigator performed daily screening for the patients potentially eligible for inclusion in the survey. The patients or their family members were contacted by the study investigators to provide written informed consent once a patient has been identified as being eligible, and then responded to the survey. The responders then completed the questionnaire survey using a desktop computer or printed form by providing anonymous scores and de-identified information. It would be expected that most of the responders would not be the patients themselves; in such cases, the investigators contacted the family members who were familiar with the patient and had the most understanding of the services provided at the ICU. It was preferred, but not mandatory that the same person responded to both surveys.

In addition to the questionnaire, the investigators also collected the following data from the patients: gender, age, admission and discharge severity scores, co-morbidities, the status of ventilator use, length of stay, and outcome. Based on our experience from the prior study [20], given a potential population size of 120 with an estimated survey score of 4.5 ± 0.65 (95% confidence interval = 4.38–4.62), we calculated the sample size to be at least 96 to reach sufficient power of 0.8 to estimate the mean score.

### Statistical analysis

We first performed a descriptive analysis of the patients and respondents regarding their demographics, clinical and social characteristics. Categorical data of the demographic, clinical, and socioeconomic features were presented as number and percentage, and continuous data were presented as mean and range. Data on SERVQUAL scores were expressed as mean and SD. The mean scores of each dimension were compared using the independent sample t-test. Mean scores of the dimensions in the survey were compared using analysis of variance (ANOVA).

The investigators plotted four-quadrant distributions using IPA [22] after having obtained the expectation and perception scales and assigned expectation and perception as the Y-axis of importance and X-axis of performance according to the relative mean scores of each item paired to the overall medians [35]. Item pairs in the upper right quadrant suggested high expectation and high perception, whereas those in the upper left quadrant suggested high expectation and low perception. The items in the lower left quadrant depicted low expectation and low perception, whereas those located in the lower right quadrant indicated low expectation but high perception. Any item located in the upper left quadrant, if found, was considered to be a priority for improvement with regards to service quality [22].

We then used binary logistic regression analysis to explore the factors associated with the gaps in service quality identified in the comparisons of survey data. Statistical analyses were performed using SPSS software (SPSS 22.0, NY, USA). A *p*-value < 0.05 was considered to be statistically significant.

## Results

### Demographic and clinical features

During the study period, 144 consecutive patients were admitted to the ICU, of whom 27 were excluded due to a lack of consent (*n* = 19), death within 24 h (*n* = 3), being transferred out within 24 h (*n* = 3), and readmission to this same ICU (*n* = 2). A total of 117 persons, including 16 admitted patients themselves and 101 members from their families, served as the respondents participating in the surveys for the on-admission expectation section; upon ICU discharge, 99 responders, including 20 patients, participated in the surveys for perception. Table 2 summarizes the demographic and clinical characteristics of the patients and the demographic and socioeconomic features of the respondents. The patients were old, more were male, and the majority (76.9%) survived to discharge from the ICU; the average length of stay at the ICU was 10.1 days. The respondents of the surveys were more commonly female (53.0%), and most were the children (39.3%) and spouse (28.2%) of the patients. Data regarding the demographics, clinical features, and questionnaire results are also available as a supplementary material (see the ‘Research Data’ file of the Supplementary Material).

**Table 2:** Demographic data and clinical features of the patients admitted to the intensive care unit (*n* = 117) and the respondents of the survey

Variable	Data
*Patients*	
Gender, male (%)	73 (62.4%)
Age (years)	65.9 (33–91)
APACHE II score on ICU admission	24.0 (5–47)
APACHE II score at ICU discharge	15.6 (5–27)
Respiratory failure on admission to the ICU	88 (75.2%)
Ventilator use on ICU discharge	30 (25.6%)
Survived to ICU discharge	90 (76.9%)
Length of stay at the ICU	10.1 (1–62)
*Respondents*	
Gender, male (%)	55 (47.0%)
Age > 65 years	52.1 (20–88)
Responder’s relation to the patient	
The patient himself or herself	16 (13.7%)
Spouse	33 (28.2%)
Child	46 (39.3%)
Sibling	8 (6.8%)
Parent	1 (0.9%)
Other	13 (11.1%)
Religion	
None	42 (35.9%)
Buddhist	40 (34.2%)
Taoist	25 (21.4%)
Christian	10 (8.5%)
An education level of college or higher	72 (61.5%)
The responder bed been living with the patient before this ICU admission	69 (59.0%)
The responder is a government employee	12 (10.3%)
The responder has a prior experience of family member admitted to an ICU	66 (56.4%)

*APACHE II* Acute physiology and chronic health evaluation II, *ICU* Intensive care unit

### Expectations and perceptions for service quality of healthcare

Table 3 shows comparisons between the perception on ICU admission and expectation scores upon ICU discharge. For the ‘tangibles’ dimension, the perception score was significantly higher than that of expectation (*p* < 0.001), whereas the scores of the two surveys were similar for the other four dimensions and the overall scores. The perception section had an overall score of 4.56 ± 0.52, and the ‘tangibles’ dimension (4.31 ± 0.63) had a significantly lower perception score among the five dimensions (ANOVA, *p* < 0.001). The other four dimensions had similar mean scores (ANOVA for the four dimensions other than ‘tangibles,’ *p* = 0.840), even though the ‘reliability’ score (4.65 ± 0.51) was the highest.

**Table 3:** Scores and gaps in the expectation and perception of service quality for each item based on the SERVQUAL scale

Expectation		Perception		gap^a^	***p***-value
Dimension/Item	Score	Dimension/Item	Score		
Tangibles	3.99 ± 0.55	Tangibles	4.31 ± 0.63	0.32 ± 0.08	< 0.001
E1	4.67 ± 0.57	P1	4.44 ± 0.70	−0.22 ± 0.09	0.011
E2	3.63 ± 0.93	P2	4.09 ± 0.78	0.46 ± 0.12	< 0.001
E3	4.12 ± 0.73	P3	4.56 ± 0.63	0.44 ± 0.09	< 0.001
E4	3.55 ± 0.89	P4	4.14 ± 0.83	0.59 ± 0.12	< 0.001
Reliability	4.73 ± 0.39	Reliability	4.65 ± 0.51	−0.08 ± 0.06	0.186
E5	4.83 ± 0.42	P5	4.68 ± 0.51	−0.15 ± 0.06	0.017
E6	4.81 ± 0.41	P6	4.68 ± 0.57	−0.14 ± 0.04	0.045
E7	4.73 ± 0.54	P7	4.64 ± 0.61	−0.09 ± 0.08	0.250
E8	4.57 ± 0.58	P8	4.63 ± 0.58	0.05 ± 0.08	0.499
E9	4.72 ± 0.57	P9	4.64 ± 0.56	−0.08 ± 0.08	0.293
Responsiveness	4.60 ± 0.43	Responsiveness	4.61 ± 0.52	0.01 ± 0.06	0.900
E10	4.58 ± 0.53	P10	4.60 ± 0.57	0.02 ± 0.08	0.844
E11	4.71 ± 0.49	P11	4.66 ± 0.57	−0.05 ± 0.07	0.467
E12	4.69 ± 0.50	P12	4.65 ± 0.54	−0.05 ± 0.07	0.518
E13	4.42 ± 0.67	P13	4.54 ± 0.59	0.12 ± 0.09	0.182
Assurance	4.66 ± 0.43	Assurance	4.64 ± 0.55	−0.02 ± 0.07	0.783
E14	4.74 ± 0.53	P14	4.68 ± 0.55	−0.06 ± 0.07	0.430
E15	4.79 ± 0.45	P15	4.68 ± 0.55	−0.11 ± 0.07	0.109
E16	4.49 ± 0.62	P16	4.60 ± 0.65	0.11 ± 0.09	0.213
E17	4.64 ± 0.59	P17	4.63 ± 0.63	−0.02 ± 0.08	0.860
Empathy	4.44 ± 0.50	Empathy	4.59 ± 0.56	0.15 ± 0.07	0.050
E18	4.36 ± 0.69	P18	4.56 ± 0.63	0.20 ± 0.09	0.030
E19	4.25 ± 0.73	P19	4.61 ± 0.60	0.34 ± 0.09	< 0.001
E20	4.35 ± 0.75	P20	4.54 ± 0.73	0.19 ± 0.10	0.069
E21	4.77 ± 0.46	P21	4.63 ± 0.60	−0.14 ± 0.07	0.049
E22	4.50 ± 0.58	P22	4.63 ± 0.56	0.13 ± 0.08	0.097
Overall	4.49 ± 0.36	Overall	4.56 ± 0.52	0.07 ± 0.06	0.219
Overall (weighted)	4.57 ± 0.81	Overall (weighed)	4.58 ± 0.52	0.01 ± 0.09	0.954

^a^gap = (perception score - expectation score)

Table 4 summarizes the assessment of the data distribution of the questionnaire. All items had a left-skewed distribution in the two surveys, with slightly higher numbers of items having a skewness number lower than − 1 in expectation (13 of 22) than in perception (9 of 22). In expectation, the dimensions of *reliability, responsiveness, and assurance* were significantly left-skewed. In perception, the dimensions of *reliability, responsiveness, assurance, and empathy* were significantly left-skewed. More items with a kurtosis were in expectation (4 of 22) than in perception (5 of 22) (Table 4). For the dimensions, kurtoses were noted in *reliability and assurance* in expectation, and *assurance* and *empathy* in perception.

**Table 4:** Assessment of data distribution for the questionnaire

Dimension/Item	Expectation		Perception	
	Skewness	Kurtosis	Skewness	Kurtosis
Tangibles	−0.182 (0.224)	−0.303 (0.444)	− 0.665 (0.243)	− 0.069 (0.481)
E1/P1	−1.558 (0.231)	1.510 (0.459)	−0.637 (0.251)	− 0.536 (0.498)
E2/P2	−0.380 (0.231)	− 0.424 (0.459)	− 0.080 (0.251)	−0.652 (0.498)
E3/P3	−0.933 (0.231)	2.115 (0.459)	−0.320 (0251)	−0.461 (0.503)
E4/P4	−0.294 (0.231)	−0.346 (0.459)	− 0.001 (0.254)	−0.858 (0.498)
Reliability	−1.937 (0.224)	4.431 (0.444)	−1.235 (0.243)	0.649 (0.481)
E5/P5	−2.562 (0.231)	6.230 (0.459)	−1.174 (0.251)	−0.636 (0.498)
E6/P6	−2.055 (0.231)	3.368 (0.459)	−1.436 (0.251)	0.898 (0.498)
E7/P7	−2.378 (0.231)	6.762 (0.459)	−0.915 (0.251)	−0.542 (0.498)
E8/P8	−1.023 (0.231)	0.066 (0.459)	−0.945 (0.251)	−0.219 (0.498)
E9/P9	−2.290 (0.231)	5.516 (0.459)	−0.199 (0.251)	1.480 (0.498)
Responsiveness	−1.072 (0.224)	0.745 (0.444)	−1.113 (0.432)	0.400 (0.481)
E10/P10	−0.777 (0.231)	−0.610 (0.459)	− 0.549 (0.251)	−1.736 (0.498)
E11/P11	−1.434 (0.231)	1.066 (0.459)	−1.031 (0.251)	−0.278 (0.498)
E12/P12	−1.497 (0.231)	1.280 (0.459)	−0.982 (0.251)	−1.060 (0.498)
E13/P13	−0.772 (0.231)	−0.552 (0.459)	− 0.765 (0.251)	−0.379 (0.498)
Assurance	−1.409 (0.224)	1.782 (0.444)	−1.422 (0.243)	1.393 (0.481)
E14/P14	−2.378 (0.231)	6.762 (0.459)	−0.651 (0.251)	0.743 (0.498)
E15/P15	−2.027 (0.231)	3.438 (0.459)	−2.104 (0.251)	3.690 (0.498)
E16/P16	−0.829 (0.231)	−0.279 (0.459)	− 0.995 (0.251)	0.013 (0.498)
E17/P17	−1.793 (0.231)	3.389 (0.459)	−1.422 (0.253)	10.79 (0.498)
Empathy	−0.503 (0.224)	−0.687 (0.444)	−1.364 (0.243)	2.256 (0.500)
E18/P18	−0.882 (0.231)	0.251 (0.459)	−0.852 (0.253)	−0.297 (0.500)
E19/P19	−0.626 (0.231)	−0.356 (0.459)	−1.130 (0.253)	0.713 (0.500)
E20/P20	−1.122 (0.231)	1.019 (0.459)	−1.052 (0.253)	0.088 (0.500)
E21/P21	−1.939 (0.231)	3.037 (0.459)	−1.369 (0.251)	0.905 (0.498)
E22/P22	−0.717 (0.231)	−0.444 (0.459)	−1.094 (0.251)	0.217 (0.498)

### Importance-performance analysis

**Fig. 1:**
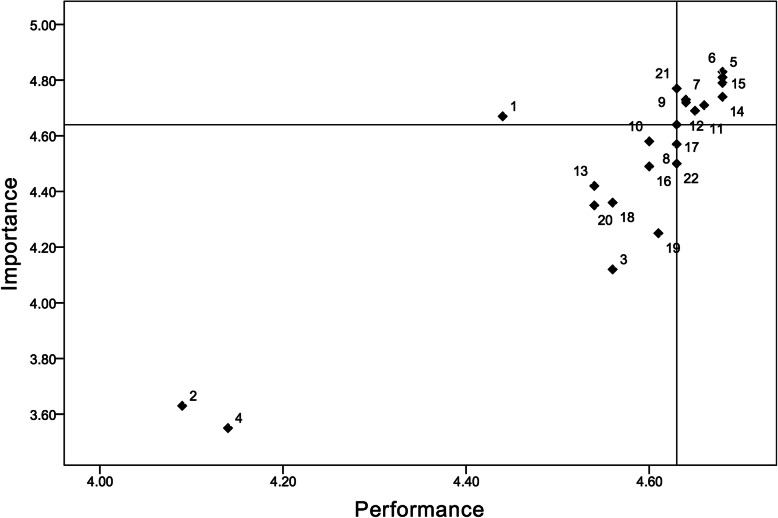
Importance-performance analysis of the expectation on admission and perception upon discharge from the ICU

Figure 1 shows the diagram of the IPA results based on the mean scores of each item by on-admission expectations (the Y-axis) and on-discharge perception (the X-axis). The diagram shows that most of the items in ‘reliability,’ ‘responsiveness’ and ‘assurance’ were located in the upper right quadrant (high expectation and high perception). In contrast, most of the items in ‘tangibles’ and ‘empathy’ were located in the lower-left quadrant (low expectation and low perception). One item (item 1) was located in the upper left quadrant (high expectation and low perception); there were no items in the right lower quadrant (low expectation and high perception) (Fig. 1).

### Factors associated with significant gaps in service quality

Table 5 summarizes the results of multivariate binary logistic regression analyses for the gaps in service quality related to ICU care. As shown in Table 3, there were no significant differences in the overall scores (4.49 ± 0.36 vs. 4.56 ± 0.52, *p* = 0.219) or weighted overall scores (4.57 ± 0.81 vs. 4.58 ± 0.52, *p* = 0.954) between expectation and perception; therefore, we decided not to perform regression analysis based on the overall score. For the ‘tangibles’ dimension, which had the lowest score for perception, most of the demographic characteristics were not associated with a *tangibles* score <  4.31 (the mean value in this survey) in perception in the multivariate analysis (*Model 1*). Further analysis including only variables with a *p*-value < 0.3 in *Model 1* revealed that ‘responder older than 65 years’ was the only factor associated with a lower dimension score (*tangibl*es score <  4.31) in perception (OR = 0.248, 95% CI = 0.091–0.678, *p* = 0.007) (*Model 2*).

**Table 5:** Multivariate logistic regression analysis for factors associated with a low *tangibles* score (< 4.31)

Item/Variable	OR	95% CI	*p*-value
*Model 1* (Hosmer-Lemeshow test: chi-square = 3.897; degree of freedom = 8; significance = 0.866)			
Patient older than 65 years	1.855	0.723–4.757	0.198
Patient was male	1.737	0.643–4.696	0.276
Patient had respiratory failure on admission to ICU	0.595	0.220–1.611	0.307
Patient was ventilator-dependent upon ICU discharge	1.917	0.623–5.899	0.256
Patient died upon ICU discharge	1.230	0.328–4.610	0.759
Responder was the patient or spouse	0.546	0.148–2.011	0.363
Responder older than 65 years	0.268	0.084–0.855	0.026
Responder had higher education (college or higher)	1.190	0.437–3.245	0.733
Responder had a religion	1.024	0.356–2.949	0.965
Responder lived with the patient	1.876	0.508–6.927	0.345
Responder was the main supporter for the patient before hospitalization	0.423	0.098–1.837	0.251
Responder was a government employee	1.681	0.379–7.454	0.494
Responder had prior experience of a family member being admitted to an ICU	1.211	0.649–2.258	0.547
Model 2* (Hosmer-Lemeshow test: chi-square = 6.581; degree of freedom = 8; significance = 0.582)			
Patient older than 65 years	2.000	0.830–4.823	0.123
Patient was male	1.426	0.577–3.527	0.442
Patient was ventilator-dependent upon ICU discharge	1.319	0.508–3.423	0.570
Responder older than 65 years	0.248	0.091–0.678	0.007
Responder was the main supporter for the patient before hospitalization	0.393	0.108–1.426	0.156

*Model 2 included only variables in Model 1 with a *p*-value < 0.3

## Discussion

In this study, to answer the research question, ‘What are the significant gaps in service quality of healthcare in the ICU?’, we used the SERVQUAL instrument to assess the service quality of healthcare in an ICU setting and understand the gaps between perception and expectation of the patients and their families. We also used IPA to explore the priorities for improvement in the service quality of healthcare. IPA showed excellent matching between performance and importance, with only one item pair of ‘tangibles’ located in the low performance-high importance quadrant. In general, the medical ICU in this study showed a performance that matched the needs perceived by the patients and their families.

Patient-centeredness has been increasingly advocated as an important domain of the assessment of healthcare performance in healthcare [36]; however, conventional satisfaction surveys lack an understanding of the expectations of the patients and their families. Although IPA has been shown to be able to assess the quality gaps and priorities for improvement [14], few reports have investigated the use of SERVQUAL and IPA in a critical care setting in Western or East Asian countries, such as quality improvement priorities in myocardial infarction care [37]. However, performance and priorities vary significantly across healthcare systems [37], and it remains unclear whether one model or instrument is better, even though other models have also been used to assess the service quality of healthcare [17, 38–40]. Our study provides further information regarding the application of these instruments and methods in healthcare services, especially the critical care setting. In addition, the ICU in this study was dedicated to caring for adult patients. The healthcare workers were not familiar with the care of patients younger than 20 years, who are mostly cared for at a children’s hospital in this healthcare system. Perceptions of responders related to younger patients may be quite different from those related to adult patients. Therefore, this study did not include any patients under 20 years of age.

Several implications of this study can be inferred. Traditionally, healthcare units use satisfaction surveys to identify areas that can be improved and target the dimensions with lower scores. In contrast, surveys using the expectation-perception model, and further deploying IPA, may provide more information regarding gaps in service quality. Therefore, the survey results may provide units with a more realistic understanding to prioritize goals to improve the service quality. The instrument used in this study was shown to have adequate validity; therefore, there is an opportunity to conduct a long-term survey with IPA and compare the results with the traditional satisfaction surveys as a research instrument for real-world healthcare service. Our data showed fair reliability and validity of the instrument with adequate sampling; therefore, we recommend the use of the SERVQUAL instrument and IPA to obtain feedback from family members in the critical care setting.

The perception of the healing environment may be important to the patient as well as the family [41]. In this study, the family members provided most of the responses to the questionnaire rather than the patients. Nevertheless, at least some of their opinions may still provide a valuable assessment of the service quality of a critical care setting. Several items, including the environment of care, the response to the need expressed by the family members, and the promise to keep planned tasks, may also be adequately reflected by family members. In Taiwan, family members are often highly involved in decision making and communication regarding the care process, and this is also shown by the high engagement in the participation in this survey by the family members. In addition, the severity of illness, indicated by the need for intubation and mechanical ventilation on admission to the ICU (about 75% of the patients), essentially precluded the possibility of eliciting valid expectation responses on admission. It has been suggested that family support may improve satisfaction in the critical care setting [42] and that the family may play an important role in patient participation in the ICU even when the patients are unable to participate [43].

Our finding that the quality gap found by SERVQUAL was not located in the upper left or lower right quadrant of IPA suggests that these two instruments may serve as complementary measures for monitoring the service quality of healthcare. A detailed understanding of unmet needs, as expressed by the patients and their families, might provide an opportunity to focus on important strategies to improve the service quality of healthcare. IPA has been advocated as a guide to improving service quality [44], and it has been suggested to be better than measuring the performance alone to predict healthcare quality and for further improvement [12]. The application of IPA has also been reported in hospitals in the East Asian region [45], and several studies that have used both SERVQUAL and IPA have been conducted in the healthcare setting [45–49], indicating a trend for greater focus on patient-orientedness. The results of SERVQUAL in this study were not compared with the results of a satisfaction survey in this unit, which was conducted periodically but not for every hospitalized patient. Therefore, we could not evaluate correlations between the measured gaps and satisfaction level regarding every dimension of the service quality of healthcare. For the ICU setting in this study, the services described in the items included in the survey questionnaire were a mixture of healthcare and administrative services, and the patients might be more likely to respond to the expectation of care and experience of received care, while the family respondents might be more likely to provide opinions about the administrative services.

In our multivariate analysis, we found that several patient and responder features were associated with quality gaps. Our finding that the older (age > 65 years) responders to the questionnaire tended to give a higher score in the *tangibles* dimension is comparable with previous studies. The outcome of death at ICU discharge was negatively associated with an increased expectation, suggesting that the family may care more about the convenience of visiting the patient when there was a favorable prognosis. Therefore, ICUs may need to respond to the need for family visits, especially when the patient has a favorable prognosis [50]. This study aimed to search for quality gaps, and this was measured by comparing perception with expectation. We found that the ‘tangibles’ dimension was the only one with a significant gap.

There are several limitations to this study. First, most (about 86%) of the responders of the surveys were family members of the patients. In addition, while there was only one responder per survey, it was not necessarily the same responder for both expectation and perception. The high likelihood of diversity of the responders and lack of paired responses may also have substantially increased the complexity of interpretation of the data. Although this was expected, as stated in the Methods section to reflect the real-world challenge in conducting surveys for ICU patients, there is a need for valid methods to understand better the expectation and perception of patients at an ICU. It is also necessary to further increase the sample size of respondents, especially the patients. Second, adequate responses to the survey questions require enough time and interaction with the healthcare workers and the care environment. However, family members may have had limited interactions in this regard. It may also be very difficult to obtain adequate responses from patients in an ICU because of their unstable physiological status, the use of sedatives and analgesics, and the use of mechanical ventilation in a bed-bound scenario. It would therefore be difficult for the patients to observe their care environment and have interactions with healthcare workers to provide adequate answers, and most of the responses may be the result of recollection. The number of responses from the hospitalized patients was limited (*n* = 16); therefore, to understand the expectations and perceptions of patients, further studies with larger patient populations are required. Similarly, most of the included patients in this study survived to discharge from the ICU, so that the family may have indicated a more satisfactory experience. Third, this study was conducted at a single center; therefore, further studies are needed to assess the generalizability of our results. Nevertheless, while we anticipate a substantial variation in the service models provided in critical care globally, even for different units within the same healthcare organization, we believe that this survey model can be generalized to other ICU settings based on its feasibility. Application of the SERVQUAL instrument in the healthcare sector remains limited, and a newer model has been developed to measure health service quality [6], which has been validated in the healthcare setting. Finally, although we applied IPA to the analysis of data obtained from SERVQUAL surveys, there is a need for a solid basis for expectation and perception of the SERVQUAL to be represented by the importance and performance of the IPA, even though there have been several reports of the combination of SERVQUAL and IPA in the area of healthcare [45–49]. Therefore, the interpretation of IPA findings should be limited to compare the expectation and perception expressed by the responders of the SERVQUAL survey. Further studies are needed to investigate and compare the practical implications of these instruments to understand the gaps in the service quality of healthcare.

## Conclusions

In conclusion, this study shows that the application of the SERVQUAL instrument and IPA might provide useful information regarding the feedback by ICU patients and their families regarding the service quality of ICU. In this study, reliable, responsive, and assured care in the ICU were the most important aspects of quality from the viewpoint of the patients and their families.

## Supplementary information

**Additional file 1.** Research Data.

## Data Availability

All data generated or analyzed during this study are included in this published article and its supplementary information files.

## Abbreviations

ICU: Intensive care unit
IPA: Importance-performance analysis
OR: Odds ratio
SERVQAL: The service quality instrument
